# Tuberculosis Treatment Compliance Under Smartphone-Based Video-Observed Therapy Versus Community-Based Directly Observed Therapy: Protocol for a Cluster Randomized Controlled Trial

**DOI:** 10.2196/38796

**Published:** 2022-07-08

**Authors:** Ponlagrit Kumwichar, Virasakdi Chongsuvivatwong, Tagoon Prappre

**Affiliations:** 1 Epidemiology Department Faculty of Medicine Prince of Songkla University Hat Yai District Thailand

**Keywords:** VOT, VDOT, video-enhanced therapy, tuberculosis, health care system, observed therapy, video-observed therapy, treatment compliance, lung disease, randomized trial, digital health, telehealth, telemedicine

## Abstract

**Background:**

The health care system in Thailand has struggled to cope with the COVID-19 pandemic, resulting in decreased administration of community-based directly observed therapy (DOT) for tuberculosis (TB). As an alternative to failed DOT, video-observed therapy (VOT) or the Thai asynchronous VOT system, “TH VOT,” was devised. We developed a protocol for a study to test the superiority of VOT over DOT in ensuring treatment compliance.

**Objective:**

We aim to compare the mean cumulative compliance days of TB patients and their observers under the VOT program with that of individuals under the DOT program during the intensive phase of TB treatment.

**Methods:**

A cluster randomized controlled trial of pulmonary TB patients and their observers will be conducted over a 2-month period. This study will be conducted in the Hat Yai and Meuang Songkhla districts of Songkhla Province, Southern Thailand. A total of 38 observers working at 38 primary care units (PCUs) will be randomized equally into VOT and DOT groups. The TH VOT system will be implemented in 19 PCUs in the VOT group while the other 19 PCUs will continue with the traditional DOT program. Approximately 1-5 TB patients will be under observation, depending on the PCU jurisdiction in which the patients reside. The inclusion criteria for TB patients will be as follows: patients diagnosed with newly active pulmonary TB with a positive acid-fast bacilli sputum smear, aged >18 years, own a smartphone, and are able to use the LINE (Line Corporation) app. The exclusion criteria will be patients with a condition that requires the intervention of a specialist, rifampicin resistance according to a cartridge-based nucleic acid amplification test (GeneXpert MTB/RIF), unable to continue the treatment, and/or alcohol dependence. After the 2-month observation period, all sessions and follow-up clinical outcomes recorded will be retrieved. An intention-to-treat analysis will be performed to assess the compliance of both patients undergoing drug administration and their observers.

**Results:**

The Human Research Ethics Committee, Faculty of Medicine, Prince of Songkla University approved the trial on February 19, 2021 (approval number 64-03618-9). The trial was funded in May 2021. The recruitment period will be from January 2022 to July 2022. The observation is scheduled to end by September 2022.

**Conclusions:**

If the VOT shows superiority in observational compliance among patients and observers, the existing DOT policy will be replaced with VOT.

**Trial Registration:**

Thai Clinical Trials Registry TCTR20210624002; https://www.thaiclinicaltrials.org/show/TCTR20210624002

**International Registered Report Identifier (IRRID):**

DERR1-10.2196/38796

## Introduction

Thailand is one of the 30 countries worldwide with the greatest tuberculosis (TB) burden [1]. During the past 2 years, Thailand has also been heavily impacted by the COVID-19 pandemic [2-4]. The pandemic led to a reduced frequency of administration of the existing community-based directly observed therapy (DOT) for TB patients and irregularity in its implementation [5-7].

Recently, a new strategy called “video-observed therapy” (VOT) was developed, which enables the provision of remote observation for patients with TB in contrast to conventional DOT. There are two forms of VOT: synchronous VOT (S-VOT) and asynchronous VOT (A-VOT) [8]. With S-VOT, observers video call their patients for real-time observation of drug administration, whereas with A-VOT, observers can review the video sent by patients at any time. Globally, A-VOT is more commonly used than S-VOT because it allows patients to record drug-administration sessions at their convenience; moreover, the video can be watched multiple times [8]. Thus, Thailand uses an A-VOT system to collect video records that can be audited [9]. Instead of a home visit in a community, an observer can flexibly use the A-VOT system to lower their travel costs and risk of SARS-CoV-2 infection.

Previous studies reported that in western countries, A-VOT has better patient compliance, cost utility, and acceptability than DOT [10-13]. However, these studies assessed A-VOT according to the number of observations of clinic-based and comprehensive community-based DOT. The irregular community-based DOT in Thailand cannot be applied to these previous studies that were conducted at an individual patient level. Owing to irregularities of DOT compliance among observers in Thailand [5], it is important to consider observation of therapy at the observer level.

To ensure an unbiased comparison between VOT and DOT, a cluster randomized controlled trial will be conducted based on a series of 2-month observations among pulmonary TB patients and their observers. A cluster randomized controlled trial design is required because in the Thai health system, patients within the same jurisdiction are observed by the same observers working at the primary care unit (PCU) of the jurisdiction.

The primary objective of this trial is to compare the mean cumulative number of compliance days of TB patients and their observers under the A-VOT program with those under the traditional community DOT program. In each jurisdiction, patient compliance with self-reporting daily drug-administration sessions and the number of doses observed will be assessed at an individual level. Patients in the same jurisdiction will represent a cluster and will be observed by the same observer. After 60 days of the intensive phase of TB treatment, the mean cumulative number of the patients’ days with self-reporting and being observed in the VOT group and the DOT group will be compared at a cluster level. The secondary objectives include performing a descriptive review of the compliance activities and comparing the clinical outcomes between patients in the VOT and DOT groups. The clinical outcomes of sputum conversion and reporting of adverse events will also be ascertained, and statistically significant differences in the secondary outcomes between the VOT and DOT groups are not expected.

## Methods

### Study Setting

This study will be conducted in the Hat Yai and Meuang Songkhla districts of Songkhla Province, Southern Thailand, where a robust internet network is available. The study setting is based on 53 PCUs in the province, each including a TB staff member who worked as a DOT observer for at least 2 years. All people in the setting are regular smartphone users.

### Background of the Existing A-VOT in Thailand

An A-VOT in Thailand (ie, the “TH VOT” system) was developed to enable remote observation of administration of anti-TB drugs taken by patients with TB [7]. The TH VOT system is a secured web system that allows patients and their observers to make a daily appointment for an observation session [14]. The system is compatible with any mobile web browsers without the requirement for installation of an additional app. A TH VOT app is also available on the Google Play store as an alternative platform that uses the system [15]. The patients and observers can easily register and use this VOT system through user authentication of the LINE (Line Corporation) app, which is the most commonly used app in Thailand [16-18]. With daily autonotification, both patients and observers will be notified about their appointment. The patients record themselves while taking their medication and upload the video to the server. The server then notifies the observer to review the video. Data regarding patient and observer compliance will be assessed according to our previous study protocol [9]. The usability of the system was tested during November 2021. It was found to function well with no technical problems and was usable among both TB patients and observers [9].

### Study Design

**Figure 1:**
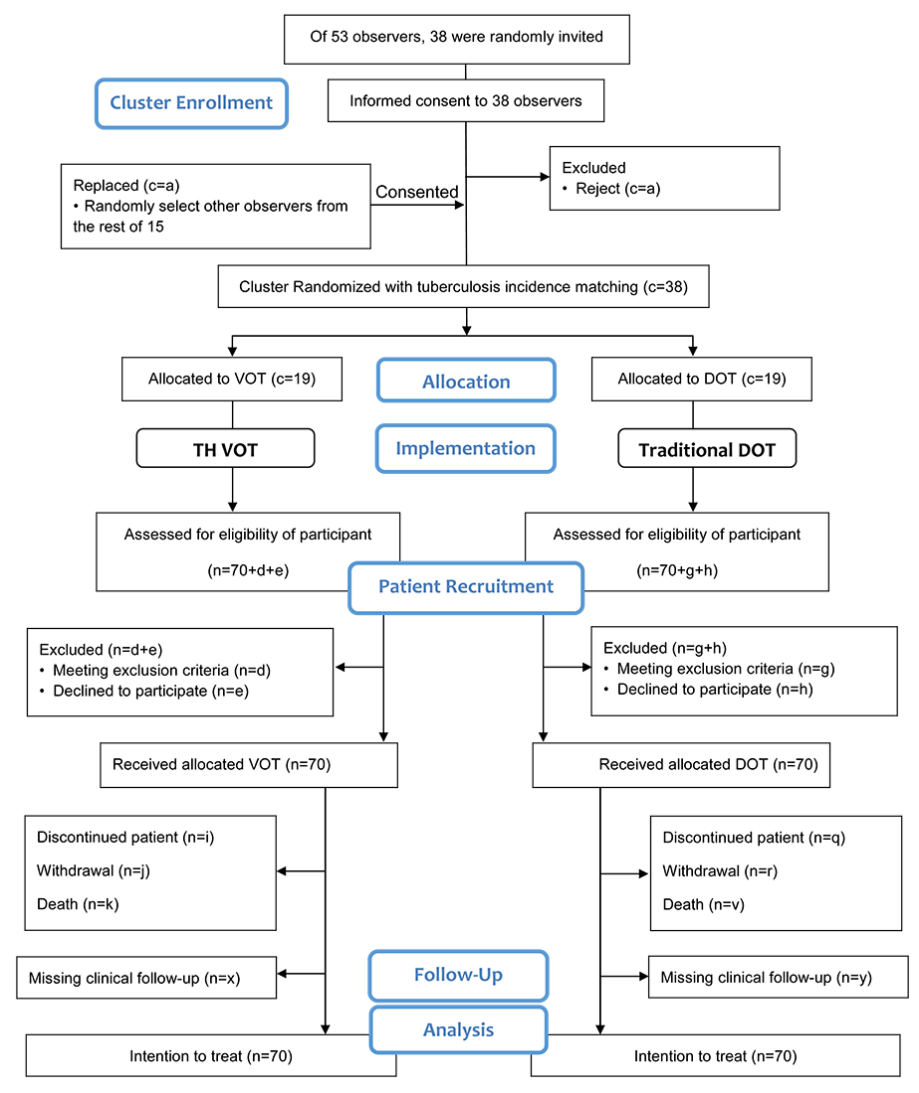
Study flow. c: number of clusters; n: number of patients with tuberculosis; DOT: directly observed therapy; TH VOT: Thailand's asynchronous video-observed therapy system; VOT: video-observed therapy.

We will perform a cluster randomized controlled trial. An observer will be assigned a cluster of patients with pulmonary TB that live in the same jurisdiction, either using DOT or VOT. We aim to observe each patient for 60 days during the intensive phase of their TB treatment. Daily compliance will be audited by one auditor who will supervise all observers in the study area according to a standard assessment protocol [9]. The study flow is summarized in Figure 1.

### Participants

#### Patients (Individual Level)

Patients will be considered eligible if they have newly active pulmonary TB diagnosed with a positive acid-fast bacilli (AFB) sputum smear, are aged>18 years, own a smartphone, and are able to use the LINE app. Participants are excluded if they have a condition that requires a specialist’s intervention, have rifampicin-resistant TB as tested by GeneXpert, unable or ineligible to continue the treatment for 60 days, or have alcohol dependence.

#### Observers (Cluster Level)

There are 53 PCUs in the study area. Based on a random list of the PCU numbers generated using R software (1-53), the observers in the PCUs numbered 1 to 38 will be randomly allocated to groups. If they refuse to undergo allocation, the observers in the PCUs numbered 39 to 53 will be subsequently invited until 38 observers have been allocated.

### Cointerventions

The following criteria will be applied to all patients and observers, regardless of their treatment group.

Patients with pulmonary TB will be diagnosed and treated by a general physician at Hat Yai Hospital or Songkhla Hospital. After diagnosis, the patients will be referred to the TB clinic. The patients will be provided zipped bags daily for 60 days, each with a daily dose of their isoniazid, rifampicin, pyrazinamide, and ethambutol (HRZE) drug regimen [7]. Patients who consent will be registered in the database by a TB nurse. The patients will be scheduled to take their medications (HRZE regimen) once a day. After each patient registration, the observer in the jurisdiction where the patient resides will be notified through autonotification on the official LINE app (either DOT or VOT).

For monetary compensation, the patients will receive 300 baht (US $8.68) immediately after registration to cover the cellular internet cost for the first month. Further compensation will be paid once the patients complete their 60-day intensive treatment without discontinuing the assigned intervention. They will receive 300 baht (US $8.68) to reimburse the cellular internet cost for the second month and 400 baht (US $11.57) for transportation of the sputum specimen for 3 consecutive days.

The observers will be compensated 600 baht (US $17.36) if they observe medication administration among patients for at least 15 out of 60 daily sessions. They will also be compensated for the cost of travel to visit their patients (4 baht or US $0.12 per kilometer).

Apart from the assigned intervention, the care of the patients at the TB clinic will remain the same as that during routine clinical practice. In the event of any suspected adverse events, the patients or their families will be able to phone the TB clinic for advice.

### Assigned Interventions

#### Individual Level

##### VOT for Patients

After registration, TB patients in the VOT jurisdiction will be trained by the TB nurse regarding how to log in to the TH VOT system through the official LINE app, and record and upload a drug-taking video session according to the standard operating procedure (SOP) [7]. Briefly, the patients must set their video frame for clear visibility of their face. All tablets and capsules should also be clearly visible. Patients should pick up the pills from the drug plane (see [7]) and put them on their tongue. Next, they should swallow the pills with clear water from a (clear) glass, raise their tongue to show the sublingual area, and stick out their tongue to show the palate area.

##### DOT for Patients

For patients in the DOT jurisdiction, the TB nurse will provide a booklet for recording daily drug intake and whether the intake is observed by the assigned observer. The TB nurse will request the patients to return the booklet and all zipped bags on the follow-up day to claim their compensation. Each weekend, the auditor will notify the patients to capture and send the most recent page of the booklet to the official LINE chat, to which the observers do not have access.

#### Cluster Level

##### VOT for Observers

To avoid a learning curve on the VOT side, the observers will be trained to demonstrate to the patient on how to correctly record and approve the recorded video according to the SOP [7]. They will be required to undergo real or simulated activities for 1 month before the trial [9].

After being notified about patient recruitment, the observers will visit patients at home on the first day. They will confirm whether the patient can record the video according to the SOP [7]. The observer and the patient will set an appointment time for medication administration. Next, the patient will keep a daily record of the drug-taking session, note any adverse events, and send the video to the observer through the TH VOT system. The observer will review the video, approve the session, and provide any necessary advice through the LINE chat box. The observer will follow up with a phone call if the patient fails to send the video within 30 minutes of the appointment.

##### DOT for Observers

Based on the guideline provided by the National Tuberculosis Information Program (NTIP) [19], which specifies that only the observer takes notes, we added an instruction regarding TB patients self-recording their daily drug-taking session.

After being notified by the automatic system, the observer will conduct home-visit DOT as routine treatment. To validate the observer’s recorded information, every weekend, the patient and the observer will be requested to take a photo of the most recent page of the booklet and send it to the auditor through the TH VOT official LINE system. The auditor will review these and record the number of daily compliant sessions in the database.

### Procedures for the Auditor to Review Each Video/Picture Session

#### Sessions in the VOT Group

“Day” will be used as the time unit for judging compliance, and local times (Greenwich Mean Time+7 hours) will be recorded. The morning will begin at 12 AM (midnight) and the evening will end at 11:59 PM. However, daily compliance will be judged as “achieved within the cut-off time” if the patients take their medication and submit their videos before 6 AM the following day. The auditor will assess daily video sessions for patients within 24 hours of the video upload. Assessment items include whether the VOT has been administered, if the patients followed the SOP, and whether they were observed as per the protocol or properly reminded by their observer. The auditor will interpret each session based on the SOP modified from the previous study [9].

The patient will be considered to have daily compliance if the video is sent within the stipulated time and if its quality meets the following criteria: (1) the patient’s face and the drug tablets/capsules are clearly visible in the video frame, (2) the pills are picked up from the drug plane and placed on the tongue, (3) the pills are swallowed with water from a (clear) glass, (4) the tongue is raised to show the sublingual area and stuck out to show the palate area, and (5) steps 2 to 4 are repeated until all pills are consumed.

The observers will be considered compliant if they adhere to the following quality standards: (1) correctly assess whether the patient performs the aforementioned steps, (2) note the number of pills consumed by the patient in the video correctly, and (3) follow up with patients via phone calls if they perform any procedure incorrectly. The TH VOT system can detect whether the observers have already made a phone call to their patients.

The daily session scored by the auditor will be recorded as 1 for daily compliance or 0 otherwise. The approval platform of the TH VOT system will automatically save all lists of the steps approved and the daily compliance score to the server database. If a patient does not record a video following the SOP, the auditor will make a phone call to remind the observer to correct the patient’s mistakes. If a patient does not send a video for 7 consecutive days, they will be considered to have discontinued the intervention.

#### Sessions in the DOT Group

The auditor will score the daily compliance weekly based on the booklet photos sent by the TB patients and their observers. The patients will be considered to have daily compliance as reported (no audit). The auditor will make a phone call to the TB patients to confirm if they were observed as reported by their observer and to remind them to safely store the booklet and all zipped bags received from the TB clinic. If the patients report that they were not observed, the auditor will further investigate and record the observer’s actions such as making phone calls instead of in-person visits to check patients’ daily drug administration, missing patient appointments, or performing activities not included in the in-person DOT protocol.

A daily session reported by the observer will be scored as 1 for daily compliance as confirmed by the patients and 0 otherwise. If a patient misses a phone call twice or loses the booklet and/or the blank zipped bags, they will be discontinued from the intervention and their daily scores of the previous 7 days will be 0. All scored sessions will be recorded in the DOT record system of the server. The activities of observers not included in the in-person DOT will also be recorded.

### Follow-ups

Each patient will be scheduled to return to the TB clinic for a follow-up on the 61st day. One day before the scheduled visit, the TB nurse will remind the patient in the DOT group to return the booklet and zipped bags. A deep-cough specimen will be collected early in the morning for 3 consecutive days. The sputum specimens will then be sent for an AFB test. The patients will be requested to notify their doctors about all adverse events occurring from the start of the treatment. The doctors will record the reported adverse events in the electronic health record (EHR) system and suggest appropriate treatment. If a patient misses their follow-up appointment, the responsible TB nurse will contact them and record the reasons in the EHR.

### Data Collection

Data regarding observational activities will be recorded in the database, and data regarding clinical outcomes will be documented in the EHR system of the involved hospitals. These records will be retrieved for analysis at the end of the follow-up.

### Outcomes

#### Primary Outcomes

Data recorded by the auditor will be compiled to understand the compliance of patients and observers in each arm. For compliance of the individual TB patients, the daily compliance scores rated by the auditor will be summed and divided by 60 (for the 60 days of observation) to obtain the percentage of cumulative days with compliance. For the whole group of patients, the mean cumulative number of compliance days will be calculated, taking the clusters into account.

Similarly, for compliance of the individual observers, the daily compliance score rated by the auditor will be calculated. The individual compliance unit of an observer will be the number of days each patient is actually observed. A higher number of patient doses observed will increase the overall contribution of the observer to the mean cumulative number of compliance days for the whole group of observers (VOT or DOT).

#### Secondary Outcomes

The clinical outcomes retrieved from the EHR, conversion of the AFB smear (three negative sputum smears as mentioned above), reporting of adverse events, missing the follow-up visit, and death during the 60-day follow-up period will be compared between the two groups.

For adverse events, information retrieved from the EHR system will be used to compare the reporting of adverse events by the observers in both the VOT and DOT groups.

### Sample Size

Each jurisdictional area comprises 1000-5000 individuals. With an approximately annual TB incidence of 130 per 100,000 individuals in Songkhla Province [20], the sample size estimate is based on the assumption that each cluster can recruit approximately 1 to 5 TB patients within 9 months.

This study is designed to detect the cumulative percentage of compliant days of the observers in the VOT group for comparison with those in the DOT group. In development of the VOT, we conducted in-depth interviews of patients with TB and their observers, and discovered that the patients had an actual appointment for DOT only once during the entire follow-up period [7]. According to our previous study [9], the cumulative percentage of compliant days among patients in the VOT group was 65%. We assumed a difference in the cumulative compliance percentage of 64% (65/100–1/60)×100. We accepted 80% statistical power and a significance level of .05 using a one-tailed test. We assumed an intracluster correlation coefficient (ICC) of 0.2. We estimated that each cluster would have approximately three patients during the study. The sample size was calculated using the group-randomized control trial calculator [21]. The variance was set to 1.00 as the default. The formula does not require an exact variance estimation in the population as the ICC takes the variance into account. The required number of clusters for each arm was 19. Thus, the number of TB patients in each group would be 19×3=57. With a sample size inflation factor of 20% to compensate for the uncertainty of TB incidence in each jurisdictional area, a sample size of 70 TB patients is required in each arm.

### Cluster Randomized Allocation

The observers that consent to participate will be randomly allocated to either VOT or DOT by a file generated using R software (R Foundation for Statistical Computing, Vienna, Austria). The sequence will be stored in the study server. Following the protocol of this trial, the participating observers will register themselves in the study LINE system. After they press the “accept” button, the observers will be informed about their allocated intervention group through the study LINE system.

### Implementation of the Trial and Patient Information

The new patients with pulmonary TB will be recruited to the VOT or DOT group by the TB nurse, depending on the jurisdiction of residence of the observer.

The relevant information regarding the study will be provided to potential patients prior to the start of the trial, including highlighting who can observe them taking their medication (ie, their observer and the auditor) along with the possible assigned interventions (VOT or DOT). The observer’s intervention group will not be revealed before they consent to participate. If the patients consent to participate, they will be assigned to the same intervention group as the observer of their PCU. They will be free to refuse the intervention at any point, and those who refuse or withdraw from the study will continue with traditional DOT without study data collection.

### Blinding

The observers will disclose their assigned intervention to the auditor, TB nurses, and researchers. Next, the researchers will train the VOT observers to familiarize them with the TH VOT system [9]; the DOT observers will be requested to perform traditional DOT as routine care. Thus, none of the researchers or staff involved in the study will be blinded to the assigned interventions.

### Statistical Analysis

Background information of patients and observers will be summarized using descriptive statistics.

An intention-to-treat analysis will be performed according to the randomized allocation. Thus, participants will be classified according to the intervention group to which they are assigned, regardless of their actual intervention.

We will compare the percentages of compliance between the two interventions at 60 days after treatment initiation. The 60-day compliance of the individuals will be defined as the percentage calculated using the following formula: (mean cumulative compliance days×100)/60. Our study is a cluster randomized controlled trial; thus, the cumulative number of compliant days of patients and observers will be nested in clusters. We will analyze their 60-day compliance considering that the same observer may monitor more than one patient. The intervention effect will be based on a linear mixed-effects model [22]. According to our study design, the intervention will be a fixed effect, whereas the cluster level will be a random effect.

The number of compliant days of patients and their observers will be visualized using solid green dots indicating the daily compliance of the individual subjects (Y-axis) and the elapsed number of days since starting the medication (X-axis). To investigate the dynamics of compliant days of the patients and observers over time, the mean cumulative number of complaint days within each group will also be plotted. The estimation of mean cumulative compliant days will range between 0 and 60, with 60 indicating that all participants in the group completed their daily sessions for 60 days. The function will be plotted using the mean cumulative number of compliant days (Y-axis) and the elapsed number of days since the start of medication administration (X-axis). Ideally, a steady increase in linear trend with a 45° slope indicates 100% compliance at any point from 0 to 60 days. Flattening of the slope represents a decline in the compliance percentage over time within the group.

Only descriptive statistical methods will be used on the aforementioned clinical outcomes and noncompliance activities of the observers because we may not have sufficient statistical power to detect small differences.

### Ethics Approval

The Human Research Ethics Committee, Faculty of Medicine, Prince of Songkla University approved the trial on February 19, 2021 (approval number 64-03618-9).

### Data Sharing

Data and programming R codes used in this trial are available in a GitHub repository [23].

## Results

This study is being supported by the Fogarty International Center and the US National Institute of Allergy and Infectious Diseases of the US National Institutes of Health under Award Number D43 TW009522 (June 14, 2021). The researchers and Deputy Province Chief Medical Officer of Songkhla Provincial Public Health Office came to an agreement on the implementation of the VOT on June 22, 2021. Recruitment started in January 2022 and will end in July 2022. The observation period is scheduled to end by September 2022.

## Discussion

### Summary

The DOT program is not sustainable in Thailand, especially during the COVID-19 pandemic period. The VOT program is a good alternative to remotely observe TB patients. The present trial will confirm whether compliance is better in observers assigned to VOT than in observers assigned to standard community-based DOT. The cluster randomized superiority trial design will minimize confounding variables and strengthen the evidence base for VOT.

### Potential Strengths

This study will assess the compliance effect on both patients and their observers in a cluster, which is more practical than using an individual effect. Compliance of both patients and observers under community-based DOT, which has rarely been studied, will be assessed in this cluster randomized superiority trial. Although treatment group blinding is not possible [24], both groups will be under the same level of monitoring by the auditor. Thus, the Hawthorne effect will be balanced.

Previous VOT versus DOT comparative studies [10,11] have been based on an overall assessment, including ordinary percentage of compliance. Our mean cumulative function analysis will determine periods with weak compliance. This can be used to reinforce observation compliance in the future.

### Limitations

Compliance of the patients in the VOT group will be directly recorded in the video records, which are accurate. However, compliance in the DOT group is only based on the patient’s report, which is not verifiable. The comparison is limited by the quality of compliance ascertainment in the DOT intervention group. The compliance of the DOT group may be overreported. If the compliance on the DOT side is lower than that on the VOT side, VOT would definitely be superior to DOT. Otherwise, the comparison may not be fully conclusive.

The main limitation is that the TB patients will not be followed up until the end of the treatment. The trial period is limited to the first 2 months of TB treatment (the intensive phase) owing to budget limitations. Sputum conversion is an uncertain surrogate of successful treatment. However, numerous studies have shown that sputum conversion is well-correlated with successful treatment [25-29].

### Conclusion

The study should provide evidence to determine whether a new policy for using VOT instead of traditional DOT for TB patients who own smartphones could improve the accountability of TB treatment monitoring. If the VOT intervention is shown to be superior to the community-based DOT intervention, we will advocate for VOT to replace the existing DOT.

## Appendix

Multimedia Appendix 1 CONSORT-eHEALTH checklist V 1.6.1.

## Abbreviations

AFB: acid-fast bacilli
A-VOT: asynchronous video-observed therapy
DOT: directly observed therapy
EHR: electronic health record
HRZE: isoniazid, rifampicin, pyrazinamide, and ethambutol
ICC: intracluster correlation coefficient
NTIP: National Tuberculosis Information Program
PCU: primary care unit
SOP: standard operating procedure
S-VOT: synchronous video-observed therapy
TB: tuberculosis
TH VOT: Thai video-observed therapy
VOT: video-observed therapy
